# El movimiento de higiene mental en Iberoamérica, 1920-1960: debates, instituciones y aplicaciones

**DOI:** 10.1590/S0104-59702025000100041

**Published:** 2025-09-15

**Authors:** Miguel Barboza-Palomino

**Affiliations:** i Profesor, Universidad Privada del Norte. Lima – Perú mbarbozapalomino@outlook.com.pe

La higiene mental fue un movimiento trasnacional impulsado principalmente por médicos psiquiatras durante la primera mitad del siglo XX. Dicho movimiento persiguió objetivos como la reforma de la atención psiquiátrica, planteó nuevas explicaciones sobre la etiología de la enfermedad mental, estimuló la creación de instituciones internacionales y nacionales de higiene mental, e incluso fue integrado como parte de proyectos políticos modernizadores implementados en diversos países.

Históricamente, la mayoría de las investigaciones sobre la aplicación de la higiene mental han centrado su atención en la América anglosajona (por ejemplo, Richardson, 1989) y países de Europa Occidental (por ejemplo, Freis, 2019). En cuanto a Iberoamérica, en las últimas décadas se ha iniciado un riguroso trabajo de indagación histórica (por ejemplo, Klappenbach, 1999) que ha analizado las particularidades que adoptó el movimiento de higiene mental en la región. En esta línea de investigación, Ricardo Campos y Mariano Ruperthuz (2022) han realizado una valiosa contribución como editores del libro *Higiene mental, psiquiatría y sociedad en Iberoamérica (1920-1960)*. Esta obra reúne cinco estudios que examinan las manifestaciones del movimiento de higiene mental en Argentina, México, Brasil, Chile y España.

En correspondencia con el número de estudios que compila el libro, este se organiza en cinco capítulos que fueron elaborados por destacados historiadores. Cada capítulo presenta un caso nacional: Hernán Scholten examina Argentina; Andrés Ríos Molina analiza México; Cristiana Facchinetti, André Mota y Pedro F. Muñiz colaboran en el estudio de Brasil; Mariano Ruperthuz, Ana Carolina Gálvez Comandini y Marcelo Sánchez Delgado ofrecen una perspectiva sobre Chile; mientras que Silvia Lévy, Ricardo Campos y Rafael Huertas muestran un análisis sobre España.

En la introducción del libro, los editores justifican la relevancia y el valor académico de la obra. Entre los argumentos que exponen se pueden destacar dos aspectos fundamentales. Primero, se presentan casos enmarcados en el creciente interés de la investigación historiográfica de la psiquiatría, la enfermedad mental y su compleja interacción con la sociedad. Segundo, los casos abordan un tema de interés actual en la investigación histórica: la diseminación y adaptación de los conceptos de higiene mental más allá de los epicentros tradicionales de Estados Unidos y Francia.

Los casos presentados revelan tanto aspectos comunes como particularidades en la aplicación del movimiento de higiene mental en Iberoamérica. Respecto al contexto donde la higiene mental toma protagonismo, destaca la noción compartida de la enfermedad mental como una epidemia que desborda los servicios de atención en salud. En algunos países, se atribuyó la enfermedad mental al crecimiento de poblaciones específicas. Por ejemplo, en Argentina se asoció con la afluencia de inmigrantes, mientras que en México se vinculó con la población pobre hacinada en las ciudades.

La higiene mental se alineó con los intereses y proyectos políticos dominantes de cada país. En Chile, esta fue incorporada a los planes de control social de los “peligrosos”, categoría que encuadraba a los enfermos mentales. En México, formó parte del proyecto de reconstrucción nacional, cuya aspiración era la formación de personas sanas para una nueva nación. En Brasil, la higiene mental fue utilizada en los discursos que estigmatizaron a los afrobrasileños pobres. En suma, estos casos también discuten y analizan las conexiones entre la higiene mental, el racismo, la teoría de la degeneración y la eugenesia.

Todos los casos analizados reportan la creación de instituciones locales de higiene mental. En Argentina, México, Brasil y España estas se denominaron ligas, mientras que en Chile llamó asociación. Un hecho en común es que estas instituciones se fundaron en el marco de la consolidación de organizaciones internacionales y la realización de eventos regionales y mundiales de higiene mental. Precisamente, los autores destacan el primero Congreso Internacional de Higiene Mental celebrado en 1930 en Estados Unidos.

En cuanto a las acciones de las ligas, se informan cuestiones similares. En concreto, estas instituciones difundieron sus actividades y contenidos sobre higiene mental a través de cartillas, boletines y revistas. Además, organizaron exposiciones fotográficas, conferencias, proyecciones de películas y documentales, jornadas informativas y programas radiales. Es importante destacar que algunas instituciones gozaron de mayor reconocimiento y apoyo que otras. Un caso notable fue la Liga Brasileña de Higiene Mental, que recibió un marcado respaldo del Estado brasileño, incluyendo un estipendio significativo para la ejecución de sus actividades.

La higiene mental incorporó ideas y prácticas gestadas por otras disciplinas. Entre estas, destaca el uso del psicoanálisis como herramienta de intervención y legitimación. En Brasil, su aplicación se enfocó en niños y trabajadores. Respecto a este último grupo, se promovía el trabajo como una actividad que ayudaba a los obreros a sublimar impulsos agresivos, fomentar la colaboración y desarrollar el autocontrol. Por su parte, en el caso español, el psicoanálisis fue utilizado como medio de legitimación social y profesional de los psiquiatras.

Los casos analizados sugieren que el desvanecimiento de la higiene mental fue paulatino y se produjo por la convergencia de varios factores. Entre estos se incluyen la disminución del protagonismo académico de los fundadores de las instituciones locales, la reducción del apoyo y subvención estatal, la implementación de nuevas formas de asistencia psiquiátrica у, principalmente, el surgimiento, promoción y consolidación del paradigma de salud mental.

En síntesis, el libro aporta información valiosa para comprender las particularidades del movimiento de higiene mental en algunos países iberoamericanos, a la vez que estimula la indagación de cuestiones aún pendientes. Sin duda, el análisis de estos casos también contribuye significativamente a revelar la trascendencia internacional y trasnacional del movimiento de higiene mental en la región.
